# Traumatic vertebrobasilar pseudoaneurysms: diagnostic pitfalls on CT angiography with forensic implications — two case reports

**DOI:** 10.1007/s10140-025-02410-w

**Published:** 2025-11-07

**Authors:** Numfon Tweeatsani, Kana Unuma, Yukiko Uemura, Hirotaro Iwase, Yohsuke Makino

**Affiliations:** 1 Department of Forensic Medicine, Graduate School of Medicine, The University of Tokyo, Tokyo, Japan; 2 Department of Forensic Medicine, Graduate School of Medicine and Dental Sciences, Institute of Science Tokyo, Tokyo, Japan; 3 Department of Legal Medicine, Graduate School of Medicine, Chiba University, Chiba, Japan

**Keywords:** CTA, Pseudoaneurysm, Trauma

## Abstract

Traumatic pseudoaneurysms of the vertebrobasilar artery are rare and may occur without skull fractures. Standard CTA may miss or misinterpret these lesions due to technical limitations and low clinical suspicion, leading to significant diagnostic and medicolegal errors; We present two cases of fatal isolated traumatic subarachnoid hemorrhage (iTSAH), where optimized thin-slice CTA, identified pseudoaneurysms of the PICA and VA (V4 segment), confirmed by histopathology.

**Graphical abstract:**

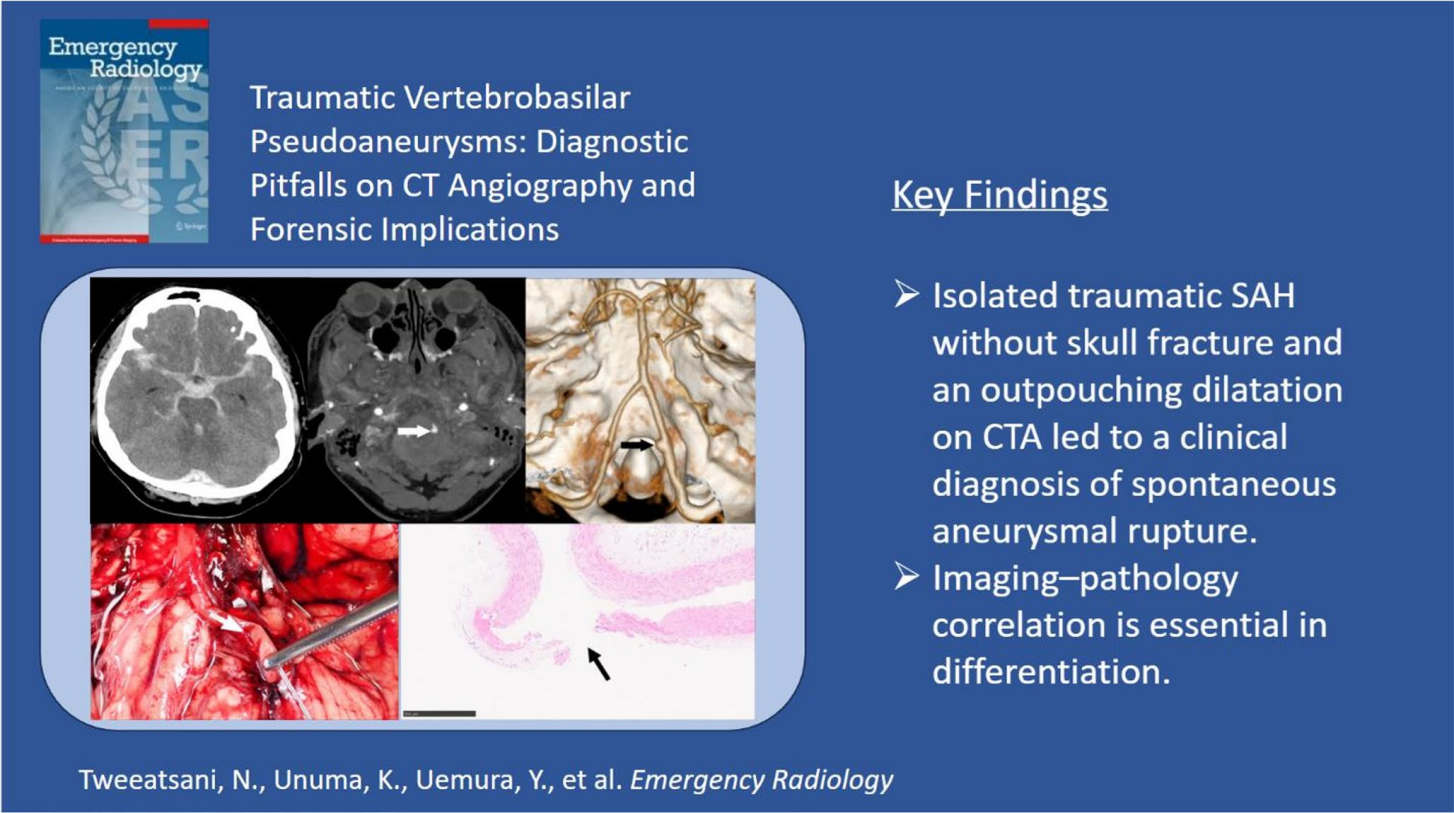

## Introduction

Traumatic pseudoaneurysms of the posterior circulation are rare but highly lethal vascular injuries following blunt head trauma [1]. They may present with isolated traumatic subarachnoid hemorrhage (iTSAH) without skull fracture or external trauma, posing diagnostic challenges. CTA is the primary diagnostic tool in emergency settings, but can have limited sensitivity due to posterior fossa artifacts, vessel tortuosity, and subtle vascular lesion [2]. Misdiagnosis as a “spontaneous” true aneurysm rupture or missed vascular injury has profound medicolegal consequences, potentially misclassifying homicide as natural death. While radiologic-pathologic correlation is essential for definitive diagnosis, literature documenting this correlation in forensic iTSAH cases remains scarce [3].

### Case 1: missed PICA pseudoaneurysm on thick-slice CTA

**Fig. 1:**
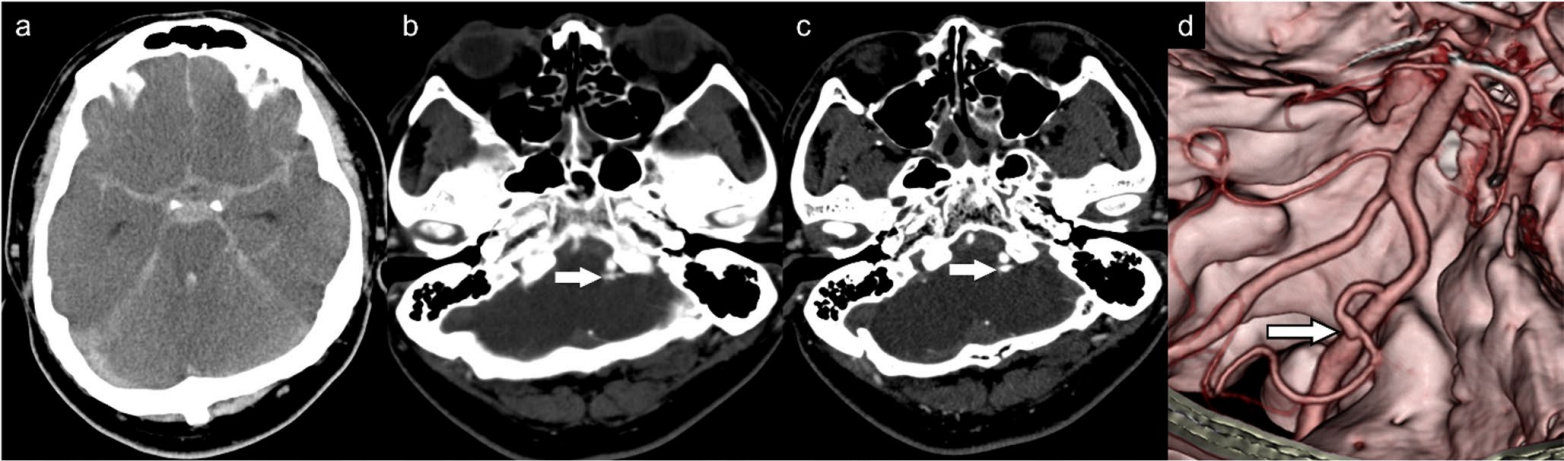
Left PICA pseudoaneurysm (**a**) NCCT shows massive SAH at the basal cistern with IVH. (**b**) 5-mm CTA fails to demonstrate a pseudoaneurysm and is misinterpreted as a non-specific vascular branch, as it was only visible in a single cut. (**c**) 0.5-mm CTA shows a focal dilatation at the proximal segment of the left PICA, visible on both axial and VRT images (**d**)

**Fig. 2:**
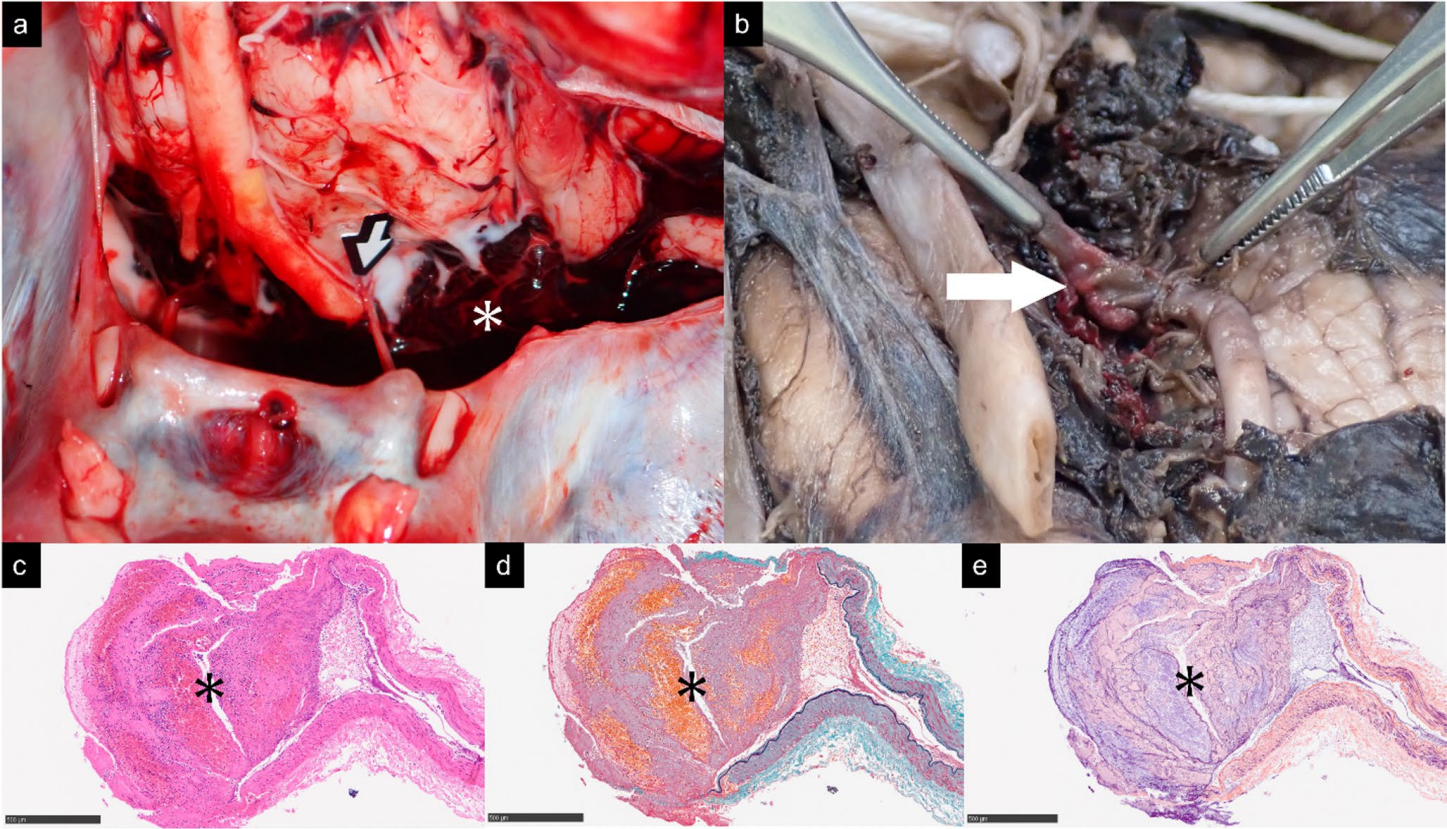
Gross autopsy and histopathologic findings. (**a**) Milk injection via the left vertebral artery shows hematoma leakage (asterisk) near the mid-V4 segment of the left VA, but it cannot identify the exact rupture site (arrow). (**b**) After fixation and clot bleaching, focal dilatation is seen at the proximal left PICA (arrow). (**c**) H&E staining shows transmural rupture with adherent clot (asterisk) and neutrophil infiltration. (**d**) EMG staining reveals no underlying vascular pathology. (**e**) PTAH staining demonstrates fibrin deposition within the clot

A man in his 50s collapsed after multiple head punches and died 59 h later. Brain CT showed massive basal SAH with IVH but no skull fracture, and initial 5-mm CTA failed to reveal a bleeding source (Fig. 1). PMCT added no further diagnostic information. Consequently, an autopsy was performed based on these preliminary imaging findings. Review of a later 0.5-mm CTA demonstrated a focal dilatation at the proximal left PICA, initially obscured on thick slices. Autopsy and histology confirmed a traumatic pseudoaneurysm with transmural rupture, fibrin deposition, and intact elastic lamina (Fig. 2).

### Case 2: misdiagnosed a VA pseudoaneurysm as a true aneurysm

**Fig. 3:**
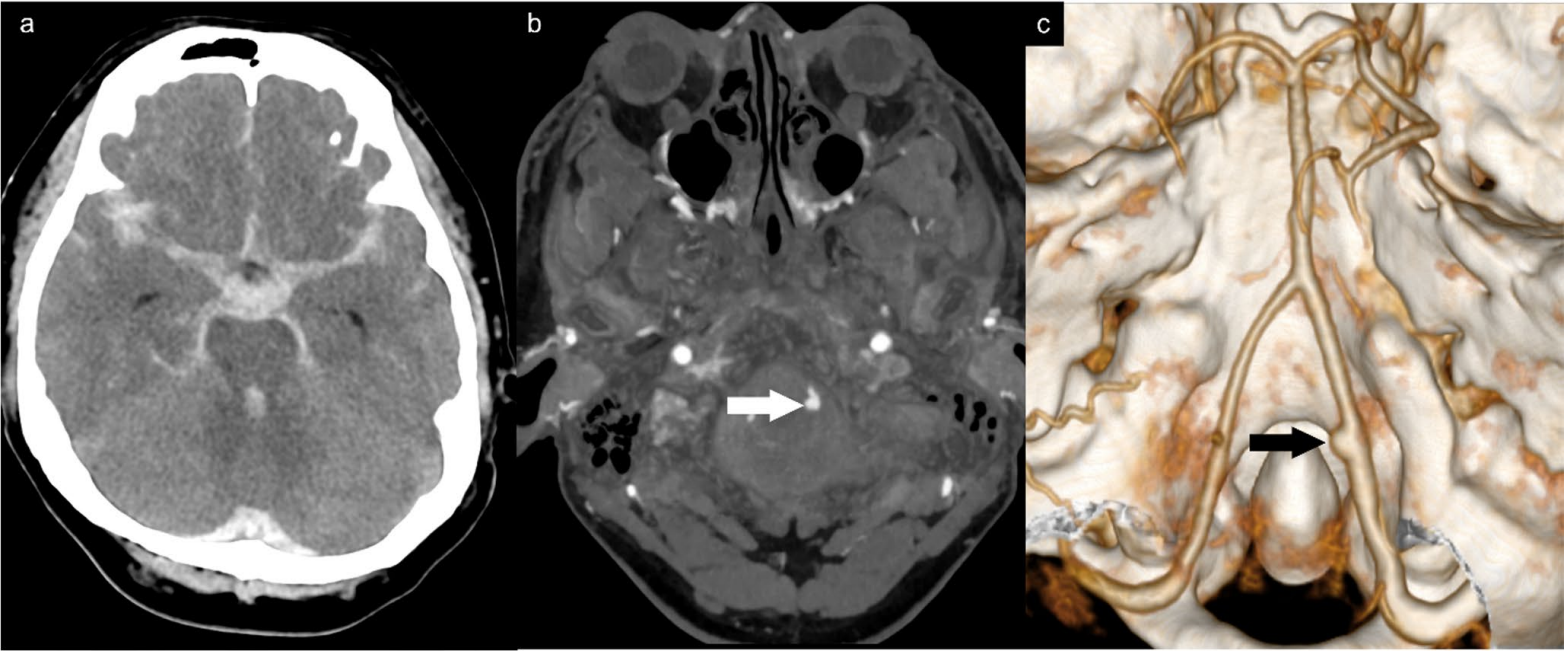
Left VA pseudoaneurysm (**a**) NCCT shows massive SAH and IVH. (**b**) 0.2-mm CTA demonstrates an outpouching vascular lesion at the medial wall of the mid-V4 segment of the left VA (arrow), which is detected on the VRT image (**c**) (arrow)

**Fig. 4:**
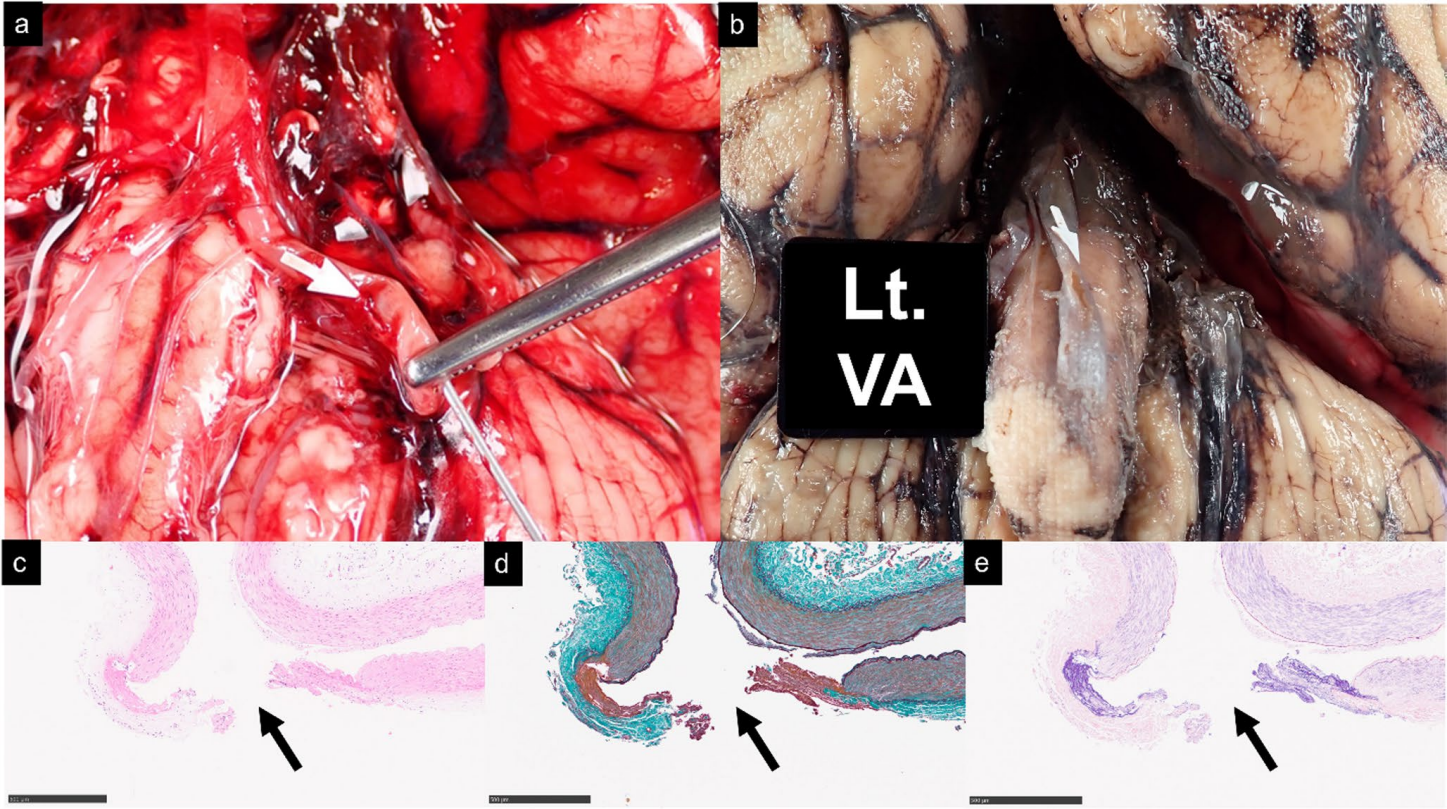
Gross autopsy and histopathology findings (**a**) A magnified view of the brain shows the rupture site and saline injection via the left VA demonstrated leakage (arrow). (**b**) After formalin fixation, the rupture site is demonstrated (arrow). (**c**) H&E staining shows complete wall disruption (arrow) with fibrin and sparse neutrophilic infiltration. (**d**) EMG staining depicts no evidence of underlying vascular pathology or inflammatory changes. (**e**) PTAH staining demonstrates fibrin deposition at the ruptured site

A man in his 40s suffered cardiac arrest after being punched multiple times during an altercation in the setting of alcohol consumption and died 54 h later after continuing resuscitation. Initial CT revealed massive basal SAH with IVH and no skull fracture (Fig. 3). CTA (2-mm slices) showed a focal outpouching at the mid-V4 segment of the left vertebral artery, interpreted clinically as a ruptured true aneurysm and certified as a natural death. Given the assault history, a forensic investigation was initiated. External exam showed left facial and right temporal contusions, and dissection revealed a 0.7-cm longitudinal tear of the left vertebral artery with leakage on saline injection. Histology confirmed complete wall rupture with fibrin deposition and acute inflammation, without evidence of preexisting vascular disease (Fig. 4).

## Discussion

The first case illustrated a fundamental limitation of conventional CTA. A PICA pseudoaneurysm was obscured on 5-mm acquisitions but identified retrospectively on 0.5-mm reconstructions with VRT. This finding supports prior study reporting pooled sensitivity was 64% (53–74%) and specificity 95% (87–99%) of CTA compared to DSA for blunt cerebrovascular injury [4]. Optimized acquisition parameters are therefore essential. ACR guideline recommends CTA with a reconstructed section thickness of 1.5 mm or less depending on the vascular territory to be assessed to maximize diagnostic yield [5]. Our findings reinforce that thin-slice CTA, combined with 3D postprocessing, is critical for detecting subtle pseudoaneurysms in the posterior circulation.

The second case emphasized the risk of misdiagnosis when imaging is interpreted without a forensic context. In this instance, basal SAH without skull fracture and an outpouching dilatation on CTA led to a clinical diagnosis of spontaneous aneurysmal rupture, and a death certificate was about to be issued as “natural death.” Autopsy findings revealed a vertical tear in the left VA, and histopathology provided definitive evidence of trauma, aligning with prior reports of trauma-induced vascular injury [6, 7]. This underscores both the absence of pathognomonic radiologic features for intracranial pseudoaneurysms [8] and the indispensable role of forensic autopsy in avoiding misclassification of cause and manner of death. Accurate distinction between traumatic pseudoaneurysms and true aneurysms is essential, as it directly impacts clinical decision-making, medicolegal classification, and attribution of responsibility. These cases demonstrate that optimized CTA protocols, cautious radiologic interpretation, and close integration with forensic pathology are crucial for reliable diagnosis in fatal traumatic SAH.

Although not applied in the present cases, postmortem CTA (PMCTA) has been proposed as a valuable adjunct when antemortem imaging is unavailable [9]. Prior studies support its utility in detecting vascular lesions that may be missed at standard autopsy, particularly in anatomically complex regions such as the skull base or posterior fossa [10, 11]. PMCTA provides a less invasive means of visualizing vascular pathology and may improve diagnostic accuracy. Future studies should evaluate its integration into forensic protocols for suspected vascular injury.

## Data Availability

All data supporting the findings of this study are available within the paper.

## Abbreviations

CTA: CT Angiography
DSA: Digital Subtraction Angiography
iTSAH: isolated traumatic subarachnoid hemorrhage
IVH: Intraventricular hemorrhage
NCCT: Non-contrast CT
PICA: Posterior inferior cerebellar artery
PMCT: Postmortem CT
PMCTA: Postmortem CT Angiography
SAH: Subarachnoid hemorrhage
VA: Vertebral artery
VRT: Virtual rendering technique
